# Psychosomatic Disorders in Patients with Gastrointestinal Diseases: Single-Center Cross-Sectional Study of 1186 Inpatients

**DOI:** 10.1155/2021/6637084

**Published:** 2021-05-01

**Authors:** Lijuan Feng, Zichun Li, Xuerong Gu, Jiahui Jiang, Xiaowei Liu

**Affiliations:** ^1^Department of Gastroenterology, Xiangya Hospital, Central South University, 410008 Changsha, China; ^2^Department of Gastroenterology and Hepatology, Shenzhen University General Hospital, 518055 Shenzhen, China

## Abstract

**Objective:**

To investigate the prevalence of anxiety and depression in hospitalized patients in the Department of Gastroenterology and to explore the risk factors affecting psychosomatic conditions in patients with digestive disorders.

**Methods:**

Patients hospitalized with gastrointestinal diseases were enrolled by the Department of Gastroenterology of Xiangya Hospital of Central South University from November 2017 to June 2018 and completed a cross-sectional questionnaire survey. According to anxiety/nonanxiety, depression/nondepression, the subjects were divided into two groups, respectively, and the risk factors of anxiety/depression were analyzed.

**Results:**

A total of 1186 patients were included in this study. The overall detection rate was 20.74% for anxiety symptoms alone, 31.78% for depressive symptoms alone, 13.99% for both anxiety and depressive symptoms, and 38.53% for either depression or anxiety symptoms. The prevalence of anxiety symptoms was higher in female than in male patients and inversely correlated with levels of education. There was no significant difference in the detection rate of anxiety and depression between patients with functional and organic digestive diseases. Sleep quality and quality of life were inversely correlated with the severity of anxiety and depression. Notably, among the patients with abnormal psychological conditions, only 7.6% of them were willing to receive psychological treatment. Gender, sleep quality, and life quality are independent risk factors for anxiety and depression symptoms for inpatients with gastrointestinal diseases.

**Conclusion:**

Paying more attention to the education level, sleep quality, and quality of life in patients with gastrointestinal diseases will help doctors to identify the risk of psychological abnormalities and improve medical care.

## 1. Introduction

As people's understanding of disease evolves from a binary regression model to a biopsychosocial medical model [1, 2], disease is deemed to be a multifactorial outcome of the interaction between psychosocial and biological factors [3]. According to the World Health Organization, anxiety and depression symptoms are the most common mental health abnormalities and have become an important source of global disease burden [4]. In recent years, anxiety and depressive disorders in patients with digestive diseases have received increasing attention. Patients with digestive disorders generally follow a long course of illness, with multiple recurrences and medical experiences. In large general hospitals, the annual number of consultations in the Department of Gastroenterology has been extremely high, and their numbers, both in- and outpatient, are one of the highest among the hospital. Previous studies have already confirmed that the comorbidity of depression and anxiety symptoms in patients with digestive disease is relatively common [5]. But unfortunately, anxiety and depression are usually perceived as risk factors for the development and progression of digestive diseases [6]. With depression or anxiety comorbidity, the physical symptoms are often aggravated, resulting in long recovery times and poor prognoses, thus consuming more medical resources [7, 8]. Although digestive diseases are closely related to mood disorders such as anxiety and depression, studies have shown that the symptoms of mood disorders in most patients with digestive diseases cannot be recognized by GI physicians [9]. As a result, 40%-90% of patients with psychological problems do not receive appropriate medical services and treatment [10, 11]. Therefore, it becomes critical to investigate and evaluate the current status of management of patients with digestive diseases with anxiety and depression. Although such studies have previously been conducted in China, more attention has been paid to certain diseases such as irritable bowel syndrome (IBS) and functional dyspepsia (FD), or the sample size was not large enough [12, 13]. The results obtained could not represent the overall prevalence of anxiety and depressive disorders and have not comprehensively analyzed the risk factors of those conditions. Our study conducted a cross-sectional survey of hospitalized patients to understand the overall incidence rate of anxiety and depression symptoms as well as explore the risk factors. It can provide a theoretical basis for screening and intervening gastroenterology patients' comorbidity with anxiety and depression.

## 2. Materials and Methods

### 2.1. Participants

Inpatients with digestive diseases treated at the Department of Gastroenterology, Xiangya Hospital of Central South University, were included in this study. The inclusion criteria were as follows:

- Admitted to the Department of Gastroenterology, Xiangya Hospital, and discharged with the first diagnosis of digestive diseases from November 2017 to June 2018
- Agree to accept the survey
- Have a sense of autonomy, have no mental retardation, and able to complete the questionnaire

The exclusion criteria were as follows:

- Unable to complete the study due to severe physical or mental illness
- Unable to complete the questionnaire

Based on these criteria, a total of 1186 patients were eventually included in the study. Informed consent was obtained from those who met the inclusion criteria, and the research design was approved by the Ethics Committee of Xiangya Hospital, Central South University.

### 2.2. Study Design and Data Collection

This is a cross-sectional study based on the Department of Gastroenterology, Xiangya Hospital of Central South University. During the period from November 2017 to June 2018, all participants were informed by the relevant medical staff (through unified training) in the ward and provided a face-to-face interview to complete the questionnaires. All the questions are mainly self-filled, while those subjects who could not complete independently were assisted by the investigators. The quality of the surveys was guaranteed by the on-site quality control method.

The questionnaire information mainly includes the following:

- General information: a self-designed general survey to investigate general patient data, including age, gender, marital status, occupation, education, and smoking and drinking history
- Self-Rating Anxiety Scale (SAS) [14]: used to determine the frequency of anxiety symptoms in the last month. The definition of SAS standard score ≥ 50 is divided into the presence of anxiety symptoms. The higher the score, the more severe the anxiety: 50 ≤ SAS < 60 for mild anxiety, 59 < SAS < 70 for moderate anxiety, and SAS > 69 for severe anxiety
- Self-Rating Depression Scale (SDS) [15]: used to assess the time frequency of depressive symptoms in the last month, and the subjects with an SDS score ≥ 53 were classified into the depression group. The higher the score, the more severe the depression: 53 ≤ SDS < 63 for mild, 62 < SDS < 73 for moderate, and SDS > 72 for severe depression
- Pittsburgh Sleep Quality Index (PSQI) Questionnaire [9]: used to assess the sleep quality of the patients in the last month. PSQI score ≤ 4 is classified into good sleep, 5-7 into sleep, and PSQI ≥ 8 into poor sleep and will be diagnosed as sleep disorders
- MOS 36-Item Short-Form Health Survey (SF-36): assesses health-related quality of life (HRQOL), which contains eight dimensions with a total of 36 items [16]. The higher the score, the better the quality of life

The diagnosis and laboratory test results of the enrolled patients were collected in the hospital medical record system, including blood routine test (CBC), liver function test, kidney function test, blood glucose and lipids, erythrocyte sedimentation rate (ESR), c-reactive protein (CRP), homocysteine (HCY), vitamins, and immune test.

### 2.3. Statistical Analysis

Statistical analysis was performed using SPSS 19.0 software. The *t*-test was used for numerical comparison between the two groups; the one-way ANOVA test was used for numerical comparison between multiple groups; the chi-square test was used to compare frequencies, and the multivariate logistic regression analysis was used for multivariate analysis.

## 3. Results

### 3.1. Overall Detection Rates of Anxiety and Depression Symptoms

Questionnaires were collected from a total of 1315 patients, and 1186 copies of the general demographic data were recovered (recovery rate: 90.19%). The overall detection rate was 20.74% (246/1186) for anxiety symptoms alone, 31.78% (377/1186) for depressive symptoms alone, 13.99% (166/1186) for both anxiety and depressive symptoms, and 38.53% (457/1186) for either depression or anxiety symptoms (Table 1).

### 3.2. Detection Rates of Anxiety and Depression Symptoms in Participants with Different Demographic Characteristics

There were 133 males and 113 females among the 246 participants with symptoms of anxiety (Table 2). Females had a significantly higher rate of anxiety than males (25.45% vs. 17.92%, *P* < 0.01). Of those patients with anxiety symptom, 71 had primary education or below (25.72% of the same academic qualifications, the same below), 91 had junior high school education (23.33%), 50 had high school education (16.78%), and 34 (15.38%) attended university. A higher rate of anxiety symptoms was detected in those with lower education levels. There were no significant differences in the rates of anxiety and depression symptoms among the other demographic characteristics of groups.

### 3.3. Detection Rates of Anxiety and Depressive Symptoms in Patients with Different Disease Types

Patients who completed the SAS and SDS questionnaire were divided into functional disease group (212 cases) and organic disease group (974 cases) according to the first diagnosis. We defined irritable bowel syndrome (IBS), functional dyspepsia, abdominal pain, and distention without severe gastrointestinal inflammation as functional disease. The organic diseases included peptic ulcer, inflammatory bowel disease (IBD), intestinal tuberculosis, colonic or rectal polyp, gastrointestinal carcinomas, autoimmune liver disease (AILD), liver cirrhosis, tuberculous peritonitis, and other more than 20 diseases.

The detection rates of anxiety symptoms in the functional disease group and the organic disease group were, respectively, 20.28%/21.04%, and the rates of depression symptoms were 29.25%/32.64% (Table 3). There was no significant difference between those two groups.

IBD patients were easy to be complicated with psychosomatic diseases. The detection rate of anxiety or depression in IBD patients was 40.7% (Table 4), which was slightly higher than the overall detection rate of 38.53%.

### 3.4. Comparison of Quality of Sleep and Life in Patients with Different Degrees of Anxiety and Depression

Among the 1315 enrolled subjects, 1143 completed the PSQI sleep scales (recovery rate was 86.92%) and 1124 completed SF-36 health-related quality of life questionnaires (recovery rate was 85.48%). Anxiety and depression were graded from mild to moderate: 50 ≤ SAS < 60 for mild anxiety, 59 < SAS < 70 for moderate anxiety, and SAS > 69 for severe anxiety; 53 ≤ SDS < 63 for mild depression, 62 < SDS < 73 for moderate depression, and SDS > 72 for severe depression. PSQI sleep scale scores and SF-36 quality of life scores were analyzed and compared in patients with each degree of anxiety or depression. The results showed that the degree of anxiety and depression was positively correlated with the increase of the PSQI score (*P* < 0.001) and was negatively correlated with the increase of the SF-36 scores (*P* < 0.001). The results are shown in Tables 5 and 6. We can draw a conclusion that the decreases in sleep quality and the decline of life quality were positively correlated with the severity of anxiety and depression.

### 3.5. Comparison of Laboratory Test Results of Anxiety/Nonanxiety and Depression/Nondepression Patients

Compared with patients without mental disorders, patients with anxiety symptoms showed elevated serum vitamin A, decreased total protein and albumin (*P* < 0.05), elevated glycated serum protein, increased CRP (*P* < 0.05), and increased aspartate aminotransferase (*P* < 0.001). Those participants with depression symptoms had significantly lower hemoglobin, albumin, and total protein (*P* < 0.01), as well as higher direct bilirubin (*P* < 0.05), high-density lipoprotein (*P* < 0.05), and aspartate aminotransferase (*P* < 0.01).

### 3.6. Analysis of Risk Factors for Anxiety and Depression

Detection of anxiety or depression was set as dependent variables. Independent variables were sex, level of education, PSQI total score, SF-36 total score, hemoglobin, serum albumin, serum total protein, high-density lipoprotein, aspartate aminotransferase, and direct bilirubin. A multivariate logistic regression model was used to analyze risk factors (Tables 7 and 8). The results are shown below: gender (*P* < 0.05), quality of sleep, and quality of life (*P* < 0.001) were independent risk factors for anxiety symptoms. Quality of sleep and quality of life (*P* < 0.001) were independent risk factors for depressive symptoms.

### 3.7. The GI Physicians' Recognition Rate of Depressive and Anxiety Disorders in Patients

245 patients among the 1186 participants were randomly selected, and their corresponding physicians were interviewed to identify whether they suffered from psychosomatic disorders before they completed the questionnaire. 79 patients were found to have anxiety/depression disorders by SAS/SDS scale; however, only 12 cases were identified by the GI specialist. The omission diagnostic rate was 84.8% (67/79), and the correct recognition rate was only 15.2%. Furthermore, 92.4% (73/79) of these patients did not agree that they had anxiety or depression disorder and were unwilling to receive corresponding psychiatric treatment.

## 4. Discussion

Patients with digestive diseases are more susceptible to symptoms of anxiety, depression, and other psychosomatic disorders. This phenomenon has a corresponding anatomical basis, that is, the regulation system of digestive function has the same anatomical position as the subcortical integration center of emotion [17]. It is necessary to investigate the psychosomatic status of patients with digestive diseases and to analyze the risk factors that affect their mental health. With a large sample size (1186 cases), this cross-sectional study investigated the prevalence of anxiety and depressive symptoms in patients with digestive diseases. Our study showed that 20.74% (246/1186) patients with digest diseases had symptoms of anxiety, which was close to the prevalence found in Europe [18] and United States [19]. The symptoms of depression appeared in 31.78% (377/1186) of patients with digestive diseases, which was higher than the prevalence (19.5%) generated from a meta-analysis of primary care patients in 10 countries [20]. This difference suggests that the incidence of anxiety and depressive symptoms may be higher in the digestive hospitalized patients than in the primary care population. In this study, the rate of detection of anxiety/depression was lower than that reported by Zhang et al. [21] and Li et al. [22]. These discrepancies may be due to the differences of screening criteria, assessment tools, and primary endpoint.

Between the two genders, the female patients were more likely to have anxiety symptoms (25.45% vs. 17.92, *P* < 0.01). Multivariate analysis further confirmed that gender was an independent risk factor for patients with anxiety symptoms, which was consistent with some previous studies [23–26]. Women are less able to regulate their emotions; one of the important reasons is their special social and functional roles [27]. In addition, the secretion levels of hormones such as sex hormones, vasopressin, and oxytocin in men and women are different, which can adjust the cerebral nervous system [28, 29]. Patients with low academic qualifications are more prone to have anxiety symptoms, and studies by Mei et al. [30] had ever reached the same conclusion. This may be because highly educated patients have a higher level of awareness of the disease state, while patients with lower education often bear greater economic pressure.

There is a two-way communication between the brain and the intestine, involving nerves, hormones, and immune pathways. Evidence from neuroscience research over the past few years has shown that the gut microbiota plays a critical role in the development and maturation of the brain system [31, 32]. We analyzed some early life events that may change the patient's gut microbiota, such as birth pattern (production/caesarean section), breastfeeding way (breastfed/not breastfed), whether full-term at birth, and whether firstborn. We set up in the questionnaire to investigate, but we did not find statistical differences. Given the possible recall bias and selection bias, we cannot completely rule out the effects of these early life events on mental health, and further forward-looking studies may lead to more reliable conclusions.

No difference in the rate of anxiety and depression was obtained between patients with organic and functional diseases of the digestive system. Many previous studies have focused on the psychosomatic status of patients with functional gastrointestinal disorders (FGIDs) such as irritable bowel syndrome (IBS) and functional dyspepsia (FD) [33–36]. It has been reported that in general hospitals, more than half of the patients with depression or anxiety visited the nonpsychiatric department for the first time. Most of them were gastrointestinal clinics, with the gastrointestinal symptoms as their first symptoms. Therefore, some gastrointestinal specialists have reached a certain consensus, when chronic patients without organic lesions came back over and over, just begin to consider their psychological factors and take suitable intervention measures and treatment [34, 37]. Our data confirm that patients with organic disease equally have serious mental health problems than those with functional disease. Recent studies have found that there is bidirectional relationship between IBD and psychological symptoms. A study from Korea enrolled 15,569 cases of IBD and 46,707 cases of non-IBD who were followed up for six years and showed that the risk of anxiety (12.2% vs. 8.7%) and depression (8.0% vs. 4.7%) was significantly increased in IBD patients [38]. Our data showed that the detection rate of anxiety or depression in IBD patients was 40.7%, higher than that of the above report, probably because the subjects in our study were hospitalized patients. Anxiety or depression can lead to negative self-management of IBD patients [39], including increasing the risk of 90-day readmission, surgery, unnecessary computed tomography, and colonoscopy. Therefore, when treating patients with organic disease, specialists should not only provide etiological treatment but also assess their mental health. Timely discovery and timely treatment can avoid the patients' psychological barriers from having great adverse effects on the development and prognosis of the disease.

In our study, patients with poor sleep quality had higher rates of anxiety and depressive symptoms. The degree of sleep disorders is positively correlated with the degree of anxiety and depression, and sleep disorders are independent risk factors for both. This observation is consistent with the findings of Hertenstein et al. [2]. Sleep disorders are found to play an important factor in the occurrence and progression of anxiety [2, 40]. Sleep can regulate the emotional function of the brain by calibrating the edge-related cortical areas of the emotions, and sleep deprivation has been shown to significantly alter activity in these specific areas [41]. A number of studies have also confirmed that sleep loss is significantly associated with depression status [42–46]. In general, mental health abnormalities are sometimes difficult to be detected by doctors, and patients with mental illness also find it hard to distinguish themselves. However, both doctors and patients can easily investigate the sleep condition. Thus, digestive specialists should actively pay attention to patients' sleep quality and realize that sleep disorders are often caused by mental illness.

Our study showed that the degree of health-related quality of life was positively correlated with the increase in the severity of anxiety and depressive symptoms. This was consistent with the findings by Gracie et al. [47, 48]. We also found that impaired quality of life is an independent risk factor for anxiety and depression. Patients with body function, social function, and physical function defect are often less independent. They mostly rely on their families and easily feel anxiety, irritability, guilt, inferiority, and depression [49]. This type of patients needs to correctly understand themselves and actively improve their own quality of life. Doctor-patient communication and family support are important ways to solve this problem.

We also separately compared the results of laboratory tests on patients with anxiety/nonanxiety and depression/nondepression, in order to find laboratory indicators associated with psychological abnormalities and to help provide theoretical basis for digestive specialists to identify patients with psychological abnormalities through laboratory indicators. By comparison, we found that patients with anxiety symptoms had elevated serum vitamin A, glycated serum protein, CRP, and aspartate aminotransferase as well as decreased serum total protein and albumin. In patients with depressive symptoms, direct bile acid and aspartate aminotransferase increased; meanwhile, serum total protein, hemoglobin, albumin, and high-density lipoprotein decreased. The overlap between the two groups was a decrease in serum total protein and albumin in patients with anxiety and depression, and this difference was more pronounced among depressed/nondepressed patients. Total protein and albumin were synthesized in the liver, and elevated transaminase also suggested that liver function may be impaired. Our results suggest that anxiety and depression may be associated with liver functions. In fact, microbiome can influence anxiety and depression via the gut-brain axis, and the linkage of gut microbiota and depression seems to be involved with the liver function. Gut bacterial microbiota and their metabolites such as lipopolysaccharides and alkaline phosphatase are common biochemical signals that occurred on the gut-liver-brain axis [50]. These bacterial metabolites can enter the liver through the portal vein, resulting in inflammation and damage in the liver [51]. Moreover, some harmful bacteria and their products could induce inflammation in the brain via blood circulation and through a cytokine cascade, thereby modulating several brain processes impacting physiological and psychological processes [16].

In addition, low protein intake or decreased protein absorption and synthesis, both of which may reduce serum total protein and albumin, and this change may indirectly lead to anxiety, depression, and other mental diseases. Our speculation depends on a view that decreased serum protein levels may lead to decreased synthesis and secretion of neuroendocrine factors such as serotonin and brain-derived neurotrophic factor (BDNF), which have been shown to link with negative emotions [52]. This may also support the possibility of brain-intestinal-liver axis regulation. In addition, some biochemical markers such as cysteine, folic acid (vitamin B9), and vitamin B12 had ever been shown to change in patients with anxiety or depression in other studies. However, no significant differences were found in our study. This may be related to differentiations in disease types and measurement tools.

In this study, the correct recognition rate of psychosomatic disorders in inpatients by Chinese digestive specialists was 15.2%, which was lower than that of nonpsychiatric doctors in the United States [53, 54]. In mainland China, a large number of patients with digestive diseases have a high comorbidity rate of anxiety and depression. But the digestive specialists' recognition rate of patients' psychological problems is very low. In addition, we also found that inpatients of the Department of Gastroenterology have low recognition of their own psychosomatic disorders and are unwilling to receive appropriate treatment. Patients often equated anxiety and depression with traditional mental disorders and felt shameful. This is one of the reasons why patients were willing to see doctors in general departments such as the gastroenterology department rather than going to a psychological counseling center or a psychiatrist. Therefore, it is necessary to strengthen the propaganda and education among patients in the daily treatment and nursing process, communicate with patients about their diseases, and strengthen psychological counseling. Continuing education for digestive specialists should also be strengthened. The existence of anxiety and depressive disorder not only affects the patients' quality of life and the therapeutic effect but also consumes a lot of medical resources. Therefore, it is very necessary to improve the ability of the digestive specialist to recognize anxiety and depressive disorder and to improve the patient's understanding of the disease and the degree of psychotherapy coordination.

In summary, our study found that among the hospitalized patients in the Department of Gastroenterology, female patients with low education, sleep disorders, nutritional disorders, and reduced quality of life were at high risk of developing anxiety and depression. GI physicians need to focus on psychological investigations of this type of patients, improve the ability to recognize psychological abnormalities in patients with digestive diseases, and take psychological or drug intervention in patients with mental disorders as early as possible to avoid some negative effects.

## Figures and Tables

**Table 1:** Overall detection rates of anxiety and depression symptoms.

Patients (*n*)	Anxiety symptoms	Depression symptoms	Depression and anxiety symptoms	Depression or anxiety symptoms
1186	246 (20.74%)	377 (31.78%)	166 (13.99%)	457 (38.53%)

**Table 2:** Detection rates of anxiety and depression symptoms in participants with different demographic characteristics.

Variable	*n* (%)	Anxiety symptoms			Depression symptoms		
		*n* (%)	*χ* ^2^	*P* value	*n* (%)	*χ* ^2^	*P* value
*Gender*			9.570	0.002		0.930	0.335
Male	742 (62.56)	133 (17.92)			229 (30.86)		
Female	444 (37.44)	113 (25.45)			148 (33.33)		
*Age (years)*			5.665	0.129		1.6996	0.637
0-17	27 (2.28)	7 (25.93)			10 (37.04)		
18-65	936 (78.92)	185 (19.76)			291 (31.09)		
66-79	202 (17.03)	52 (25.74)			70 (34.65)		
>79	21 (1.77)	2 (9.52)			6 (28.57)		
*Marital status*			3.801	0.283		0.586	0.899
Married	1067 (89.97)	220 (20.62)			340 (31.87)		
Unmarried	101 (8.52)	21 (20.79)			31 (30.69)		
Divorced	18 (1.51)	4 (22.22)			6 (33.33)		
*Education*			13.174	0.019		5.052	0.168
Primary/below	276 (23.27)	71 (25.72)			98 (35.51)		
Junior high	391 (32.88)	91 (23.33)			131 (33.59)		
High	298 (25.12)	50 (16.78)			87 (29.19)		
College/above	221 (18.63)	33 (14.93)			61 (27.60)		
*Profession*			7.571	0.476		10.541	0.229
Civil servant/staff	245 (20.66)	42 (17.14)			78 (31.84)		
Worker	98 (8.26)	22 (22.45)			34 (34.69)		
Farmer	305 (25.72)	67 (21.97)			112 (36.72)		
Student	43 (3.63)	8 (18.60)			13 (30.23)		
Retirement	156 (13.15)	32 (20.51)			46 (29.49)		
Freelancer/unemployed	339 (28.58)	75 (22.12)			94 (27.72)		
*Smoking*			1.214	0.270		0.048	0.826
Yes	362 (30.52)	68 (18.78)			116 (32.04)		
No	824 (69.48)	178 (21.60)			261 (31.67)		
*Drinking*			3.267	0.070		1.406	0.235
Yes	273 (23.02)	46 (16.85)			78 (28.57)		
No	913 (76.98)	200 (21.91)			299 (32.75)		

**Table 3:** Detection rates of patients' anxiety and depression symptoms with different diseases.

Disease type	*n* (%)	Anxiety symptoms			Depression symptoms		
		*n* (%)	*χ* ^2^	*P* value	*n* (%)	*χ* ^2^	*P* value
Functional disease	212 (26.31)	43 (20.28)	**0.014**	**0.907**	62 (29.25)	**1.109**	**0.292**
Organic disease	974 (73.69)	205 (21.04)			318 (32.64)		

**Table 4:** Detection rate of anxiety or depression in IBD patients.

	Number *n*	Anxiety *n* (%)	Depression *n* (%)	Anxiety or depression *n* (%)
IBD	108	29 (26.9)	37 (34.3)	44 (40.7)
CD	77	23 (29.9)	26 (33.8)	32 (41.6)
UC	31	6 (19.4)	11 (35.5)	12 (38.7)

**Table 5:** PSQI scores in different degrees of anxiety and depression.

PSQI	Normal	Anxiety			*P* value	Normal	Depression			*P* value
		Mild	Moderate	Severe			Mild	Moderate	Severe	
*n*	906	172	45	20		784	223	115	23	
Sleep quality	1.30	1.82	1.97	2.40		1.28	1.71	1.73	2.21	
Sleep latency	1.27	1.93	1.90	2.17		1.23	1.82	1.72	2.02	
Sleep time	1.75	2.23	2.28	2.80		1.75	2.04	2.20	2.26	
Sleep efficiency	1.98	2.43	2.75	2.95		1.98	2.25	2.50	2.60	
Sleep disorder	2.27	2.68	2.84	3.45		2.26	2.58	2.61	3.13	
Hypnotic	0.08	0.25	0.17	0.25		0.10	0.12	0.18	0.39	
Daytime function	1.34	1.98	2.38	2.80		1.28	1.91	2.03	2.43	
*Total score*	**7.03**	**10.36**	**11.33**	**13.82**	**<0.001**	**6.91**	**9.46**	**9.98**	**12.06**	**<0.001**

**Table 6:** SF-36 scores in different degrees of anxiety and depression.

SF-36	Normal	Anxiety			*P* value	Normal	Depression			*P* value
		Mild	Moderate	Severe			Mild	Moderate	Severe	
*n*	894	167	44	19		776	213	111	24	
Physical function	78.23	66.23	62.95	45.26		79.59	68.33	65.00	45.21	
Body role function	12.33	6.70	7.57	1.32		12.80	7.75	7.68	2.63	
Physical pain	62.94	49.93	43.43	23.26		64.07	51.29	50.19	30.92	
General health	56.67	48.65	47.95	38.68		57.83	49.60	46.94	40.83	
Energy	83.14	63.78	54.36	37.32		85.78	67.22	56.88	37.08	
Social function	81.87	64.51	59.93	36.42		84.12	66.22	62.52	40.33	
Social affective role	15.63	8.56	9.84	3.89		16.30	10.34	8.68	4.13	
Mental health	83.80	65.75	57.39	47.63		85.39	71.95	5959	46.88	
*Total score*	**474.59**	**374.11**	**343.43**	**233.79**	***<0.001***	**458.88**	**392.69**	**357.49**	**248.02**	***<0.001***

**Table 7:** Logistic regression of anxiety symptom-related factors in patients.

	*B*	S.E.	Wals	*P*	OR	95% CI
Gender	0.373	0.189	3.876	0.049	1.452	1.002-2.104
Education	-0.113	0.103	1.212	0.271	0.893	0.730-1.092
Hemoglobin	0.006	0.003	2.764	0.096	1.006	0.999-1.013
Total protein	-0.010	0.017	0.362	0.548	0.990	0.958-1.023
Albumin	0.001	0.019	0.004	0.947	1.001	0.964-1.040
LDL	0.552	0.286	3.726	0.054	1.736	0.992-3.040
AST	0.002	0.001	2.534	0.111	1.002	1.000-1.004
PSQI score	0.112	0.023	24.256	<0.001	1.119	1.070-1.170
SF-36 score	-0.006	0.001	47.118	<0.001	0.994	0.992-0.996
Constant	-0.582	1.023	0.323	0.570	0.559	

**Table 8:** Logistic regression of depression symptom-related factors in patients.

	*B*	S.E.	Wals	*P*	OR	95% CI
Gender	-0.057	0.170	0.112	0.726	0.942	0.676-1.314
Education	0.014	0.090	0.024	0.884	1.013	0.850-1.207
Hemoglobin	0.000	0.003	0.011	0.924	1.000	0.994-1.006
Total protein	-0.004	0.014	0.071	0.777	0.996	0.968-1.025
Albumin	-0.005	0.017	0.072	0.778	0.995	0.963-1.029
LDL	0.147	0.258	0.324	0.616	1.136	0.689-1.873
AST	0.000	0.001	0.027	0.954	1.000	0.998-1.003
PSQI score	0.007	0.001	12.452	<0.001	1.074	1.032-1.118
SF-36 score	-0.071	0.020	77.224	<0.001	0.993	0.991-0.994
Constant	2.020	0.893	5.121	0.024	1.002	

## Data Availability

We provide all the underlying data in the tables of the manuscript.
